# Evaluation of a deep learning software for automated measurements on full-leg standing radiographs

**DOI:** 10.1186/s43019-024-00246-1

**Published:** 2024-11-29

**Authors:** Louis Lassalle, Nor-Eddine Regnard, Marion Durteste, Jeanne Ventre, Vincent Marty, Lauryane Clovis, Zekun Zhang, Nicolas Nitche, Alexis Ducarouge, Jean-Denis Laredo, Ali Guermazi

**Affiliations:** 1 Réseau Imagerie Sud Francilien, Lieusaint, France; 2 Ramsay Santé, Clinique du Mousseau, Evry, France; 3 Gleamer, Paris, France; 4 Service de Radiologie, Institut Mutualiste Montsouris, Paris, France; 5 Laboratoire (B3OA) de Biomécanique et Biomatériaux Ostéo-Articulaires, Faculté de Médecine Paris-Cité, Paris, France; 6 Université Paris-Cité, Paris, France; 7 Department of Radiology, Boston University School of Medicine, Boston, MA USA

**Keywords:** Leg measurements, Artificial intelligence, Radiography

## Abstract

**Background:**

Precise lower limb measurements are crucial for assessing musculoskeletal health; fully automated solutions have the potential to enhance standardization and reproducibility of these measurements. This study compared the measurements performed by BoneMetrics (Gleamer, Paris, France), a commercial artificial intelligence (AI)-based software, to expert manual measurements on anteroposterior full-leg standing radiographs.

**Methods:**

A retrospective analysis was conducted on a dataset comprising consecutive anteroposterior full-leg standing radiographs obtained from four imaging institutions. Key anatomical landmarks to define the hip–knee–ankle angle, pelvic obliquity, leg length, femoral length, and tibial length were annotated independently by two expert musculoskeletal radiologists and served as the ground truth. The performance of the AI was compared against these reference measurements using the mean absolute error, Bland–Altman analyses, and intraclass correlation coefficients.

**Results:**

A total of 175 anteroposterior full–leg standing radiographs from 167 patients were included in the final dataset (mean age = 49.9 ± 23.6 years old; 103 women and 64 men). Mean absolute error values were 0.30° (95% confidence interval [CI] [0.28, 0.32]) for the hip–knee–ankle angle, 0.75 mm (95% CI [0.60, 0.88]) for pelvic obliquity, 1.03 mm (95% CI [0.91,1.14]) for leg length from the top of the femoral head, 1.45 mm (95% CI [1.33, 1.60]) for leg length from the center of the femoral head, 0.95 mm (95% CI [0.85, 1.04]) for femoral length from the top of the femoral head, 1.23 mm (95% CI [1.12, 1.32]) for femoral length from the center of the femoral head, and 1.38 mm (95% CI [1.21, 1.52]) for tibial length. The Bland–Altman analyses revealed no systematic bias across all measurements. Additionally, the software exhibited excellent agreement with the gold-standard measurements with intraclass correlation coefficient (ICC) values above 0.97 for all parameters.

**Conclusions:**

Automated measurements on anteroposterior full-leg standing radiographs offer a reliable alternative to manual assessments. The use of AI in musculoskeletal radiology has the potential to support physicians in their daily practice without compromising patient care standards.

## Background

Evaluation of the alignment, joint orientation, and lengths of the lower limbs on anteroposterior full-leg standing radiographs plays a pivotal role in understanding musculoskeletal health. For example, varus and valgus deformities are recognized as a major risk factor for knee osteoarthritis [1–3]. Moreover, leg length discrepancy is often associated with vestibular dysfunction and spinal alterations [4]. Accurate radiographic assessment of lower limb alignment not only is critical for mitigating these risks but also forms a cornerstone of effective surgical planning in high tibial osteotomy or knee arthroplasty [5–7].

Numerous parameters of the lower extremities can be measured on anteroposterior full-leg standing radiographs to diagnose varus and valgus knees or leg length discrepancy [8, 9]. These include the hip–knee–ankle (HKA) angle, pelvic obliquity, leg lengths, femoral lengths, and tibial lengths. In clinical practice, they are often performed manually or with interactive software applications [10]. Despite their widespread use, these approaches have been criticized for their time-consuming and labor-intensive nature [10], and for occasionally lacking consistency and accuracy. The variability in results is compounded by the absence of a universal consensus on the placement of landmark points [11, 12]. Moreover, manual radiographic measurements have been shown to be affected by the experience of the reader [13].

Fully automated solutions could minimize the challenges posed by more traditional measurement methodologies by providing standardized analysis and reproducible measurements of radiographic images. The Data Science Institute of the American College of Radiology highlights the importance of automating lower limb measurements. They list leg length discrepancy as one of the use case scenarios for which artificial intelligence (AI) holds the potential to improve medical care [14]. The latter exemplifies the increased interest in AI in the realm of musculoskeletal imaging [15–17]. AI-based solutions have been shown to aid in the detection of fractures [18–20], to increase the precision of bone age assessment [21, 22], or to predict the progression of osteoarthritis [23, 24]. The automation of skeletal measurements has garnered increased attention, and software have been developed for the spine [25, 26], the hip [27, 28], the foot [29, 30], and the leg [31–33]. Worthy of note, both conventional radiography and advanced imaging modalities such as EOS imaging can be used for such measurements [34].

This study aimed to compare the performance of a European Conformity (CE)-certified commercially available AI solution (BoneMetrics, Gleamer, Paris, France) to the manual measurements of expert musculoskeletal radiologists on anteroposterior full-leg standing radiographs. The assessment focused on estimating a comprehensive array of lower limb angles and lengths from conventional radiography and EOS imaging. Secondary objectives were to compare AI performance across these modalities and evaluate differences on the basis of the use of distinct landmark points. It was hypothesized that BoneMetrics would achieve accuracies comparable to the reference standard.

## Methods

The study received institutional review board approval for the retrospective collection of patient data from four institutions (ethics approval number CRM-2209-306). Each institution informed patients about the use of their anonymized data for research purposes, and provided instructions on how to opt out of the study.

### Dataset

Radiographs were selected from a database aggregating data from four radiology private practices in France, spanning a period from January 2015 to October 2019. Radiologists’ reports were queried in the database to identify radiographs that had been acquired for leg measurements using a natural language processing (NLP) algorithm. The latter method involved using regular expressions (“pangonogram,” “goniometry,” “measurement(s),” “angle(s),” and “degree(s)”) to identify phrases related to lower limb measurements from text. Data on participant sex were extracted from the Digital Imaging and Communication in Medicine (DICOM) tags. Inclusion criteria were anteroposterior full-leg standing radiographs, acquired with conventional radiography or EOS imaging, from patients over 3 years old. Radiographs with orthopedic implant were deemed eligible. A sample of 296 radiographs met the inclusion criteria. An expert musculoskeletal radiologist (N.R.) with 13 years of experience reviewed the dataset and excluded radiographs for one of the following reasons: visible measurements on the image, poor image quality (e.g., blurry, under-exposed, or containing a grid), incorrect patient positioning obscuring key anatomical details, rotational issues, or non-weight-bearing X-rays.

### Measurement definitions

This study focused on a comprehensive set of leg and hip measurements including the hip–knee–ankle (HKA) angle, pelvic obliquity, leg lengths, femoral lengths, and tibial length. Each measurement was performed separately on the left and right legs, with the exception of pelvic obliquity. In total, there were seven measurements of interest. The HKA angle was defined as the angle between the line drawn from the center of the femoral head to the center of the femoral intercondylar notch and the line drawn from the center of the tibial spines to the center of the distal tibia [8, 9]. Pelvic obliquity was computed as the distance between the horizontal line passing through the top of the right femoral head and the horizontal line passing through the top of the left femoral head and a vertical line [35]. Pelvic obliquity can only be measured on radiographs for which both right and left legs are analyzable. Two distinct leg lengths were defined according to the position of the landmark point on the femoral head. The “top leg length” and “center leg length” were computed as the axes running from the top of the femoral head to the center of the distal tibia and from the center of the femoral head to the center of the distal tibia [36]. Similarly, two distinct femoral lengths were defined as a function of the position of the landmark point on the femoral head. The “top femoral length” and the “center femoral length” were defined as the axes extending from the top of the femoral head to the center of the femoral intercondylar notch and from the center of the femoral head to the center of the femoral intercondylar notch [36]. Finally, tibial length was outlined as the distance from the center of the tibial spines to the center of the distal tibia [36].

### Manual ground truth measurements

Two expert musculoskeletal radiologists with 13 and 12 years of experience carried out manual measurements independently (N.R. and L.L.). Radiographs were presented on a dedicated web-based data annotation platform (Kili) equipped with an array of labeling tools such as zoom, pan, contrast adjustment, and a circle drawer. Annotators placed 10 landmarks on each image: the top of the left and right femoral heads, the center of the left and right femoral heads, the center of the left and right femoral intercondylar notches, the center of the left and right tibial spines, and the center of the left and right distal tibiae. In cases where hip or knee implants were present, the radiologists placed the landmarks on the basis of identifiable prosthesis features, such as the top or the center of the prosthetic femoral head and the well-defined midpoint of the femoral and tibial components of the knee implant. These landmarks allowed the measurements to be computed for each leg. The ground truth was defined as the mean of the measurements provided by both radiologists. To evaluate intra-reader reliability, one of the two expert musculoskeletal radiologists (N.R.) re-annotated a random sample of 28 anteroposterior full-leg standing radiographs from the dataset after a 1-month washout period.

### Automated AI measurements

This study tested the BoneMetrics AI-based software (version 2.3.1, Gleamer, Paris, France). BoneMetrics is a CE-marked image processing tool that automates musculoskeletal measurements on conventional radiographs and EOS images. The software consists of multiple convolutional neural networks that allow for the detection and localization of landmark points and the subsequent computation of measurements. The algorithm predicts landmark points with a confidence score from 0 to 100, and only points with scores exceeding the predefined threshold of 50 are used to calculate the set of measurements. BoneMetrics relies on varied architectures including a top-down model implemented with detectron2, a lightweight HRNet (litehrnet), and a bottom-up approach. Such diversity strengthens the flexibility and robustness of the AI across a wide array of radiographic images.

The algorithm was trained on a dataset comprising more than 5000 images from 20+ European imaging centers, and included patients with and without implants. The radiographs were labeled by 10 radiographers and radiologists who had received prior training. All annotations were reviewed by an expert musculoskeletal radiologist with 14 years of experience to ensure optimal quality of the training dataset. No radiograph or patient included in the present study was used for the development of the AI.

### Statistical analysis

The sample sizes for this study were based on calculations from a previous similar study [32]. They computed the sample sizes necessary to compare AI measurements with ground truth values for various parameters of interest, including the HKA angle, leg length, femur length, tibial length, and pelvic obliquity. Their computations were guided by a method for Bland–Altman analysis, which requires the clinically meaningful maximum difference allowed [37]. At an alpha of 0.05 and a power of 0.8, they found the required sample sizes to be 16 legs for the HKA, 10 legs for leg length, 8 legs for femur length, 22 legs for tibial length, and 60 legs for pelvic obliquity.

Summary statistics were computed for patient characteristics (sex and age) and for image characteristics (modality and center of origin). Analyses were conducted independently for each leg except for pelvic obliquity, which was computed at the patient level. Performance of the AI algorithm was evaluated using the root mean square error (RMSE) and mean absolute error (MAE), with 95% confidence intervals (CI) calculated using 1000 bootstrap resamples. To address data dependencies from multiple radiographs per patient and multiple measurements per radiograph (left and right), patient resampling was used for all parameters but pelvic obliquity. The MAE was also computed for radiographs of pediatric versus adult patients and patients with and without hip or knee implant and for conventional radiography versus EOS imaging. Additionally, the MAE for the HKA was compared between patients with and without lower limb malalignment, genu varum and genu valgum being characterized by an HKA angle differing by 3° or more from neutral alignment [38, 39]. Differences between these groups were assessed using the Mann–Whitney *U* test for pelvic obliquity and linear mixed models for all other measurements with patient as a random effect to account for dependencies within the dataset. Statistical significance was set at *p* = 0.007 (= 0.05/7 measurements) for the children versus adults, knee implant versus no implant, and conventional radiography versus EOS analyses and at *p* = 0.008 (= 0.05/6 measurements) for the hip implant versus no implant after adjusting for multiple comparisons using the Bonferroni method.

Agreement between the AI software and the ground truth was assessed using Bland–Altman analyses. For pelvic obliquity, the traditional Bland–Altman method was applied. For all other parameters, a mixed-effects approach to the Bland–Altman analysis was used, with the patient as a random effect and both radiograph and laterality as fixed effects. In addition, intraclass correlation coefficients (ICC) were calculated to assess AI and ground truth agreement and intra-reader variability, on the basis of two-way mixed-effects models with absolute agreement, as well as inter-reader variability, on the basis of a two-way random-effects model with absolute agreement. ICC values were classified as poor (ICC < 0.5), moderate (0.5 ≤ ICC < 0.75), good (0.75 ≤ ICC < 0.9), or excellent (ICC ≥ 0.9), according to Koo et al. (2016) [40]. All statistical computations were performed using R (v4.3.2) in RStudio (v2023.09.1 + 494) with the “irr” and “blandr” packages.

## Results

### Dataset characteristics

**Fig. 1:**
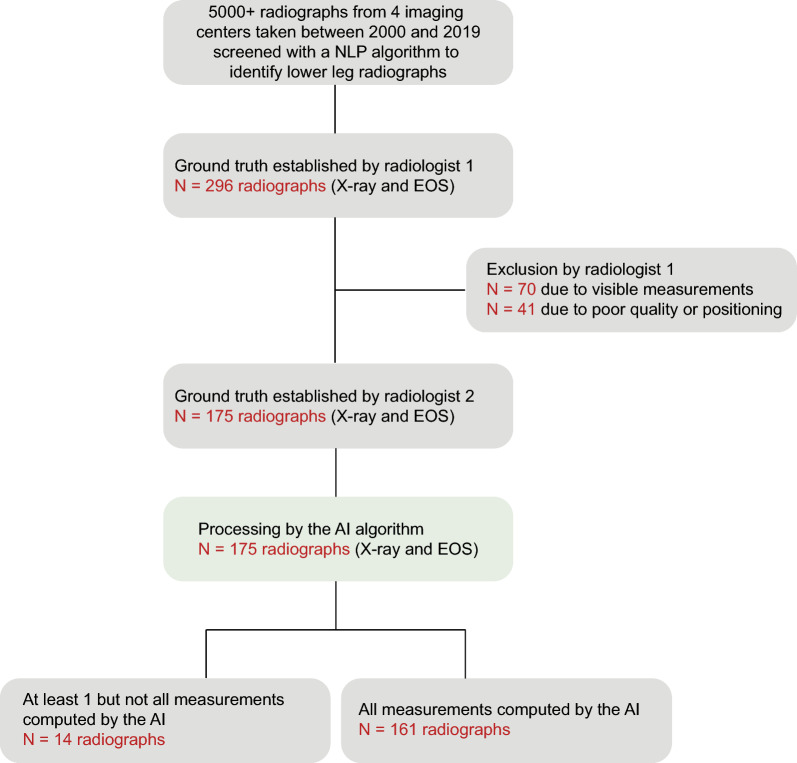
Flowchart describing dataset selection, data processing, and final available measurements

The reviewing phase by the expert musculoskeletal radiologist led to the exclusion of 111 images: 41 due to poor quality and 70 due to measurements being visible on the radiograph (Fig. 1). The final dataset included in the study comprised 175 anteroposterior full-leg standing radiographs from 167 patients, including 129 conventional radiographs (73.7%) and 46 EOS images (26.3%). Eight patients had two radiographs taken at different time points. The distribution of radiographs across center was as follows: 17 images from Center 1 (9.7%), 22 images from Center 2 (12.6%), 106 images from Center 3 (60.6%), and 30 images from Center 4 (17.1%). There were 107 radiographs from 103 female patients (61.1%) and 68 radiographs from 64 male patients (38.9%). The mean age of patients was 50 years (standard deviation = 24), and ages ranged from 3 to 89 years (Table 1). Among the 26 pediatric patients included in the study, 10 patients still had open growth plates.

**Table 1:** Characteristics of patients included in the dataset

	Patients	Radiographs
Sample size (*n*)	167	175
Age		
Mean ± SD (years)	49.9 ± 23.6	
Range (years)	[3.1–89.0]	
Number of children	26	
Sex		
Women (%)	103 (61.7%)	107 (61.1%)
Men (%)	64 (38.3%)	68 (38.9%)
Imaging modality		
Conventional radiography (%)	121 (72.5%)	129 (73.7%)
EOS (%)	46 (27.5%)	46 (26.3%)
Orthopedic implant		
Hip prosthesis (%)	22 (13.2%)	24 (13.7%)
Knee prosthesis (%)	38 (22.8%)	38 (21.7%)
Malalignment		
Unilateral genu varum (%)	45 (26.9%)	48 (27.4%)
Bilateral genu varum (%)	38 (22.8%)	39 (22.3%)
Unilateral genu valgum (%)	16 (9.6%)	17 (9.7%)
Bilateral genu valgum (%)	4 (2.4%)	4 (2.3%)
Leg length discrepancy (%)	22 (13.2%)	22 (12.6%)

Within the dataset, there were 57 patients with an orthopedic implant (34.1%) including 19 patients with a hip prosthesis (11.4%), 35 with a knee prosthesis (21.0%), and 3 with both (1.8%). Regarding malalignment, unilateral genu varum (HKA angle > 3°) was identified in 45 patients (26.9%), while bilateral genu varum was observed in 38 patients (22.8%). Unilateral genu valgum (HKA angle < −3°) was present in 16 patients (9.6%) and bilateral genu valgum in 4 patients (2.4%). Finally, leg length discrepancy, defined as a difference greater than 10 mm between the left and right legs, was noted in 22 patients (13.2%; Table 1). Among these, six patients had orthopedic implants (27.3%).

### AI performance and agreement with ground truth

**Fig. 2:**
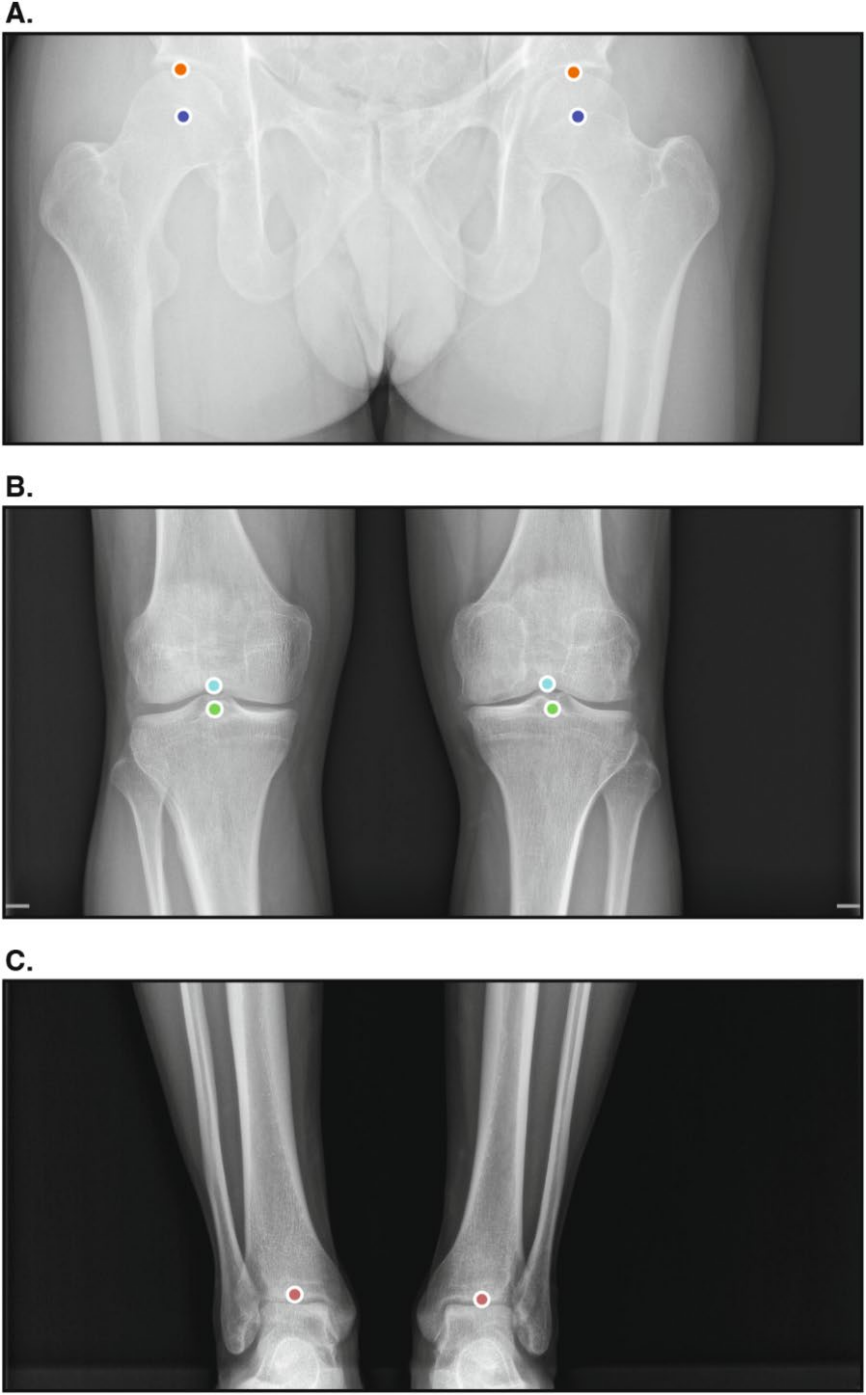
Illustrations of the landmark points placed by the annotators who established the ground truth on a dedicated web-based platform. There were five unique landmarks for each leg. **A** The orange point corresponds to the top of the femoral head and the purple point to the center of the femoral head. **B** The light blue point corresponds to the femoral intercondylar notch and the green point to the center of the tibial spine. **C** The red point corresponds to the center of the distal tibia

In total, 350 legs were available from 175 radiographs. Figure 2 provides illustrations of how the radiologists who established the ground truth placed the landmark points. There were 163 missing measurements from 28 radiographs; the missing measurements were thus excluded from subsequent analyses (Table 2). Pelvic obliquity measurements were missing in the presence of a hip prosthesis, as the AI software does not yield any output in such instances. Most of the other missing parameters were also observed in patients with a hip prosthesis due to low AI confidence scores (< 50%).

**Table 2:** Number of legs for which a measurement was not computed by the AI

Lengths and angles	Legs without measurement/total (%)
HKA	19/350 (5.4%)
Pelvic obliquity	25/175 (7.1%)
Top leg length	41/350 (11.7%)
Center leg length	18/350 (5.1%)
Top femoral length	38/350 (10.9%)
Center femoral length	16/350 (4.6%)
Tibial length	6/350 (1.7%)

**Fig. 3:**
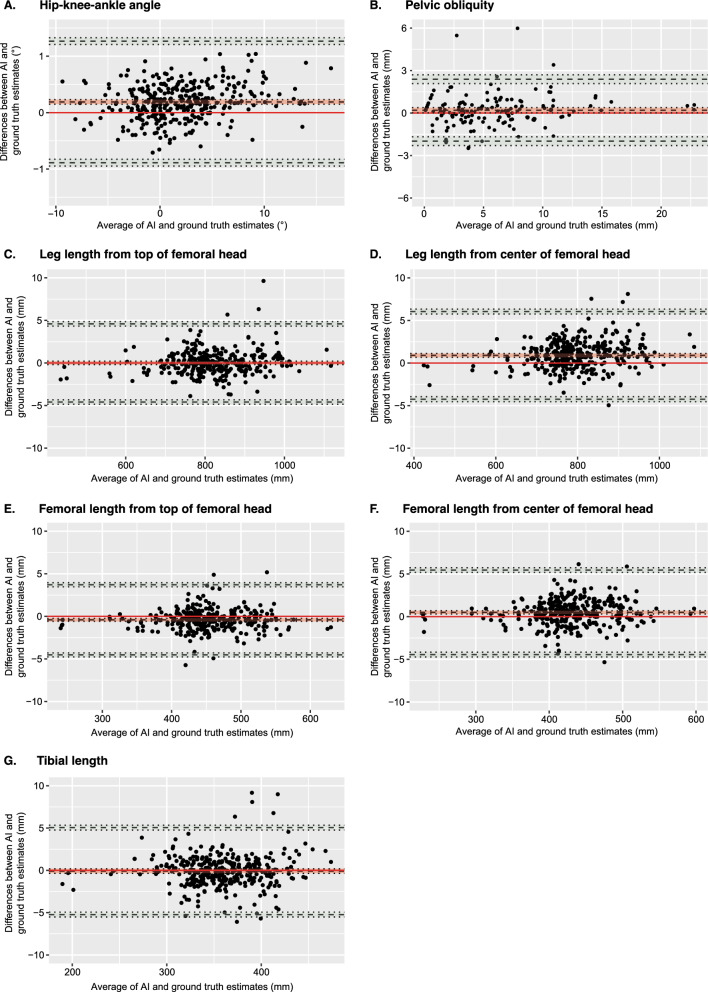
Bland–Altman plots showing the differences between AI and ground truth predictions against their means. Plots are displayed for hip–knee–ankle angle (**A**), pelvic obliquity (**B**), leg length measured from the top of the femoral head (**C**), leg length from the center of the femoral head (**D**), femoral length measured from the top of the femoral head (**E**), femoral length measured from the center of the femoral head (**F**), and tibial length (**G**). The red line depicts the scenario in which AI estimates would perfectly align with the ground truth, indicating no differences between the two. The light red interval around the black dotted line corresponds to the 95% confidence interval for the mean difference. The black dotted lines at the extremities of the plot represent the upper and lower limits of agreement with their respective 95% confidence intervals in light green. For all measurements but pelvic obliquity, a mixed effects Bland–Altman analysis was used to account for dependencies within the dataset

RMSE and MAE for each measurement are displayed in Table 3, and results of the Bland–Altman analyses are shown in Table 4 and Fig. 3. RMSE was smallest for the HKA angle (0.37°, 95% CI [0.34, 0.39]) and pelvic obliquity (1.13 mm, 95% CI [0.87, 1.30] and largest for the tibial length (2.02 mm, 95% CI [1.60, 2.29]). MAE was also smallest for the HKA angle (0.30°, 95% CI [0.28, 0.32]) and pelvic obliquity (0.75 mm, 95% CI [0.64, 0.86]), but it was largest for the center leg length (1.46 mm, 95% CI [1.34, 1.57]). Regarding Bland–Altman analyses, the HKA angle and pelvic obliquity demonstrated small biases of 0.19° (95% CI [0.15, 0.23]) and 0.20 mm (95% CI [0.019, 0.38]), respectively, with narrow limits of agreement. Top leg length and tibial length exhibited near-zero biases of 0.004 mm (95% CI [−0.19, 0.20]) and −0.087 mm (95% CI [−0.36, 0.18]), respectively. In contrast, center leg length, top femoral length, and center femoral length displayed higher biases, with the center leg length showing the highest bias at 0.90 mm (95% CI [0.68, 1.11]). Notably, all leg measurements were associated with wider limits of agreement (Table 4).

**Table 3:** Performance of the AI algorithm for each measurement

Lengths and angles	Sample size (*N*)	RMSE [95% CI]	MAE [95% CI]
HKA (°)	331	0.37 [0.34, 0.39]	0.30 [0.28, 0.32]
Pelvic obliquity (mm)	150	1.13 [0.87, 1.30]	0.75 [0.64, 0.86]
Top leg length (mm)	309	1.45 [1.19, 1.61]	1.03 [0.92,1.13]
Center leg length (mm)	331	1.89 [1.68, 2.04]	1.46 [1.34, 1.57]
Top femoral length (mm)	312	1.29 [1.14, 1.42]	0.95 [0.86, 1.04]
Center femoral length (mm)	334	1.57 [1.44, 1.69]	1.23 [1.13, 1.32]
Tibial length (mm)	344	2.02 [1.60, 2.29]	1.38 [1.21, 1.53]

Performance was assessed by root mean square error (RMSE) and mean absolute error (MAE). In addition, 95% confidence intervals were computed using bootstrapping with patient resampling to account for data dependencies. Sample size (N) is also displayed

**Table 4:** Results of the Bland–Altman analyses for each measurement of interest

Lengths and angles	Sample size (*N*)	Bias [95% CI]	Lower LOA [95% CI]	Upper LOA [95% CI]
HKA (°)	331	0.19 [0.15, 0.23]	−0.89 [−0.95, −0.83]	1.27 [1.21, 1.33]
Pelvic obliquity (mm)	150	0.20 [0.019, 0.38]	−1.99 [−2.30, −1.68]	2.39 [2.08, 2.69]
Top leg length (mm)	309	0.004 [−0.19, 0.20]	−4.59 [−4.85, −4.33]	4.60 [4.33, 4.86]
Center leg length (mm)	331	0.90 [0.68, 1.11]	−4.25 [−4.53, −3.97]	6.04 [5.76, 6.32]
Top femoral length (mm)	312	−0.41 [−0.57, −0.25]	−4.53 [−4.76, −4.29]	3.71 [3.47, 3.94]
Center femoral length (mm)	334	0.50 [0.32, 0.69]	−4.45 [−4.72, −4.17]	5.45 [5.18, 5.72]
Tibial length (mm)	344	−0.087 [−0.36, 0.18]	−5.23 [−5.51, −4.95]	5.06 [4.78, 5.33]

For all measurements but pelvic obliquity, a mixed-effects approach was applied to account for data dependencies. Patient was modeled as a random effect, while radiograph and laterality of the measurement were treated as fixed effects. Sample size (N), bias, lower limit of agreement (LOA), upper LOA and their 95% confidence intervals (CI) are displayed

The ICC between AI predictions and the ground truth was excellent (≥ 0.97) for all seven measurements (Table 5). Top leg length and top femoral length had the lowest RMSE (1.13 mm, 95% CI [0.87, 1.30]; 1.29 mm, 95% CI [1.14, 1.42]) and MAE (1.03 mm, 95% CI [0.92, 1.13]; 0.95 mm, 95% CI [0.86, 1.04]), suggesting that these parameters deviated less from the ground truth. In contrast, center leg length and center femoral length showed slightly higher RMSE (1.89 mm, 95% CI [1.68, 2.04]; 1.57 mm, 95% CI [1.44, 1.69]) and MAE (1.46 mm, 95% CI [1.34, 1.57]; 1.23 mm, 95% CI [1.13, 1.32]), underlining greater deviation from the ground truth.

**Table 5:** Results of the agreement analyses

Lengths and angles	Sample size (*N*)	ICC between AI and GT [95% CI]	Intra-reader reliability [95% CI]	ICC between radiologists [95% CI]
HKA	331	> 0.99 [> 0.99, 1]	> 0.99 [> 0.99, 1]	> 0.99 [0.99, 1]
Pelvic obliquity	150	0.97 [0.96, 0.98]	0.98 [0.95, 0.99]	0.99 [0.98, 0.99]
Top leg length	309	> 0.99 [> 0.99, 1]	> 0.99 [> 0.99, 1]	> 0.99 [0.95, 1]
Center leg length	331	> 0.99 [> 0.99, 1]	> 0.99 [> 0.99, 1]	> 0.99 [> 0.99, 1]
Top femoral length	312	> 0.99 [> 0.99, 1]	> 0.99 [> 0.99, 1]	> 0.99 [> 0.99, 1]
Center femoral length	334	> 0.99 [> 0.99, 1]	> 0.99 [> 0.99, 1]	> 0.99 [> 0.99, 1]
Tibial length	344	> 0.99 [> 0.99, 1]	> 0.99 [> 0.99, 1]	> 0.99 [> 0.99, 1]

Agreement between AI and the ground truth (GT) was assessed with intraclass correlation coefficients (ICC) from a two-way mixed-effects model with absolute agreement for multiple raters. The intra-reader reliability was assessed with ICC from a two-way mixed-effects model with absolute agreement for a single rater. Agreement between the two radiologists who established the ground truth was evaluated with ICC from a two-way random-effects model with absolute agreement for multiple raters. Sample size (N) is also displayed

The performance of the AI was compared between pediatric and adult patients, and no statistically significant differences were noted for any parameter (Table 6). To evaluate the influence of hip and knee implants on the accuracy of AI measurements, separate analyses were conducted for radiographs of patients with and without implants (Table 6). Statistically significant differences were found between patients with and without hip implants for the algorithm’s estimation of the center leg length (*p* = 0.006) and center femoral length (*p* < 0.001). Patients with hip implants (1.25 mm, 95% CI [0.97, 1.49]) had lower MAE values than those without for the center leg length (1.46 mm, 95% CI [1.30, 1.60]). In contrast, patients with hip implants (1.43 mm, 95% CI [1.17, 1.66]) had higher MAE values than those without for the center femoral length (1.18 mm, 95% CI [1.07, 1.29]). Significant differences were also noted in the accuracy of tibial length estimates (*p* < 0.001), with a MAE of 1.82 mm (95% CI [1.44, 2.16]) in the presence of a knee implant compared with 1.15 mm (95% CI [1.01, 1.28]) without implant. The HKA angle was the only parameter for which the accuracy of AI measurements was significantly affected by imaging modality (*p* < 0.001; Table 6). Measurements on conventional radiographs had a MAE of 0.32° (95% CI [0.30, 0.24]), while those on EOS images had a significantly smaller MAE of 0.25° (95% CI [0.22, 0.28], *p* < 0.001). To assess the impact of malalignment on the HKA angle, measurements for patients with genu varum or genu valgum were compared with those of patients with neutral alignment (Table 6). The MAE for patients with genu varum was significantly higher than that for patients without (*p* < 0.001), with respective values of 0.35° (95% CI [0.32, 0.39]) and 0.30° (95% CI [0.28, 0.32]). No significant differences were found in the estimation of the HKA angle between patients with and without genu valgum (*p* = 0.28).

**Table 6:** Comparison of the performance of the AI algorithm across different groups

Lengths and angles	MAE [95% CI]		Statistical test
	Children (*N* = 26)	Adults (*N* = 141)	
HKA (°)	0.27 [0.22, 0.31]	0.31 [0.28, 0.33]	F(1, 162) = 6.00, *p* = 0.02
Pelvic obliquity (mm)	0.58 [0.43, 0.72]	0.79 [0.66, 0.91]	W = 1365, *p* = 0.15
Top leg length (mm)	0.87 [0.75, 0.99]	1.07 [0.94, 1.17]	F(1, 155) = 1.51, *p* = 0.22
Center leg length (mm)	1.13 [0.96, 1.30]	1.53 [1.38, 1.65]	F(1, 162) = 1.31, *p* = 0.25
Top femoral length (mm)	0.73 [0.62, 0.83]	1.00 [0.89, 1.09]	F(1, 156) = 0.01, *p* = 0.92
Center femoral length (mm)	0.90 [0.74, 1.04]	1.29 [1.19, 1.39]	F(1, 163) = 0.00, *p* = 0.99
Tibial length (mm)	1.22 [0.96, 1.47]	1.41 [1.20, 1.58]	F (1, 164) = 3.81, *p* = 0.05

	Hip implant (*N* = 19)	No implant (*N* = 110)	
HKA (°)	0.26 [0.21, 0.30]	0.30 [0.27, 0.32]	F(1, 127) = 4.50, *p* = 0.04
Pelvic obliquity (mm)	NA	NA	NA
Top leg length (mm)	0.74 [0.56, 0.93]	1.01 [0.88, 1.12]	F(1, 120) = 0.014, *p* = 0.91
Center leg length (mm)	1.25 [0.97, 1.49]	1.46 [1.30, 1.60]	F(1, 127) = 7.82, p = 0.006*
Top femoral length (mm)	0.83 [0.43, 1.16]	0.89 [0.80, 0.98]	F(1, 121) = 0.034, *p* = 0.85
Center femoral length (mm)	1.43 [1.17, 1.66]	1.18 [1.07, 1.29]	F(1, 128) = 11.49, p < 0.001^*^
Tibial length (mm)	1.84 [1.04, 2.50]	1.15 [1.02, 1.29]	F(1, 129) = 1.60, *p* = 0.21

	Knee implant (*N* = 35)	No implant (*N* = 110)	
HKA (°)	0.33 [0.29, 0.36]	0.30 [0.27, 0.32]	F(1, 142) = 0.098, *p* = 0.75
Pelvic obliquity (mm)	1.04 [0.69, 1.33]	0.66 [0.57, 0.75]	W = 2173, *p* = 0.48
Top leg length (mm)	1.16 [0.99, 1.34]	1.01 [0.88, 1.12]	F(1, 142) = 0.030, *p* = 0.86
Center leg length (mm)	1.57 [1.36, 1.78]	1.46 [1.30, 1.60]	F(1, 142) = 0.12, p = 0.72
Top femoral length (mm)	1.16 [0.94, 1.36]	0.89 [0.80, 0.98]	F(1, 143) = 0.68, *p* = 0.41
Center femoral length (mm)	1.28 [1.10, 1.45]	1.18 [1.07, 1.29]	F(1, 143) = 0.049, *p* = 0.83
Tibial length (mm)	1.80 [1.48, 2.07]	1.15 [1.02, 1.29]	F(1, 142) = 34.30, *p* < 0.001^*^

	Conventional radiographs (*N* = 121)	EOS images (*N* = 46)	
HKA (°)	0.32 [0.30, 0.34]	0.25 [0.22, 0.28]	F(1, 162) = 25.22, *p* < 0.001^*^
Pelvic obliquity (mm)	0.80 [0.65, 0.93]	0.58 [0.45, 0.72]	W = 1788, *p* = 0.32
Top leg length (mm)	1.04 [0.91, 1.16]	1.01 [0.86, 1.15]	F(1, 155) = 5.40, *p* = 0.02
Center leg length (mm)	1.54 [1.40, 1.68]	1.26 [1.10, 1.41]	F(1,162) = 2.43, *p* = 0.12
Top femoral length (mm)	0.97 [0.87, 1.08]	0.89 [0.72, 1.04]	F(1, 156) = 2.55, *p* = 0.11
Center femoral length (mm)	1.24 [1.14, 1.35]	1.19 [1.05, 1.33]	F(1, 163) = 1.05, *p* = 0.31
Tibial length (mm)	1.44 [1.22, 1.63]	1.22 [1.06, 1.38]	F(1, 164) = 0.67, *p* = 0.41

	Genu varum (*N* = 83)	All (*N* = 167)	
HKA (°)	0.33 [0.30, 0.36]	0.30 [0.28, 0.32]	F(1, 162) = 14.79, *p* < 0.001^*^

	Genu valgum (*N* = 20)	All (*N* = 167)	
HKA (°)	0.28° [0.22, 0.34]	0.30 [0.28, 0.32]	F(1, 162) = 1.18, *p* = 0.28

Performance of the AI algorithm on pediatric and adult patients, on images with and without implant (knee and hip prosthesis), on conventional radiographs versus EOS images, and on images with and without malalignment for the HKA angle, as assessed by the mean absolute error (MAE). Mann–Whitney *U* tests were computed to evaluate how implant and imaging modality influenced differences between AI-based and ground truth measurements of pelvic obliquity. Linear mixed models with patient as a random effect were employed to assess the influence of implant and imaging modality on all measurements but pelvic obliquity. Additional linear mixed models with patient as a random effect were computed to examine the influence of genu varum and genu valgum on differences between AI and ground truth measurements. Counts represent the number of patients rather than the number of images.

^*^Statistically significant result

**Fig. 4:**
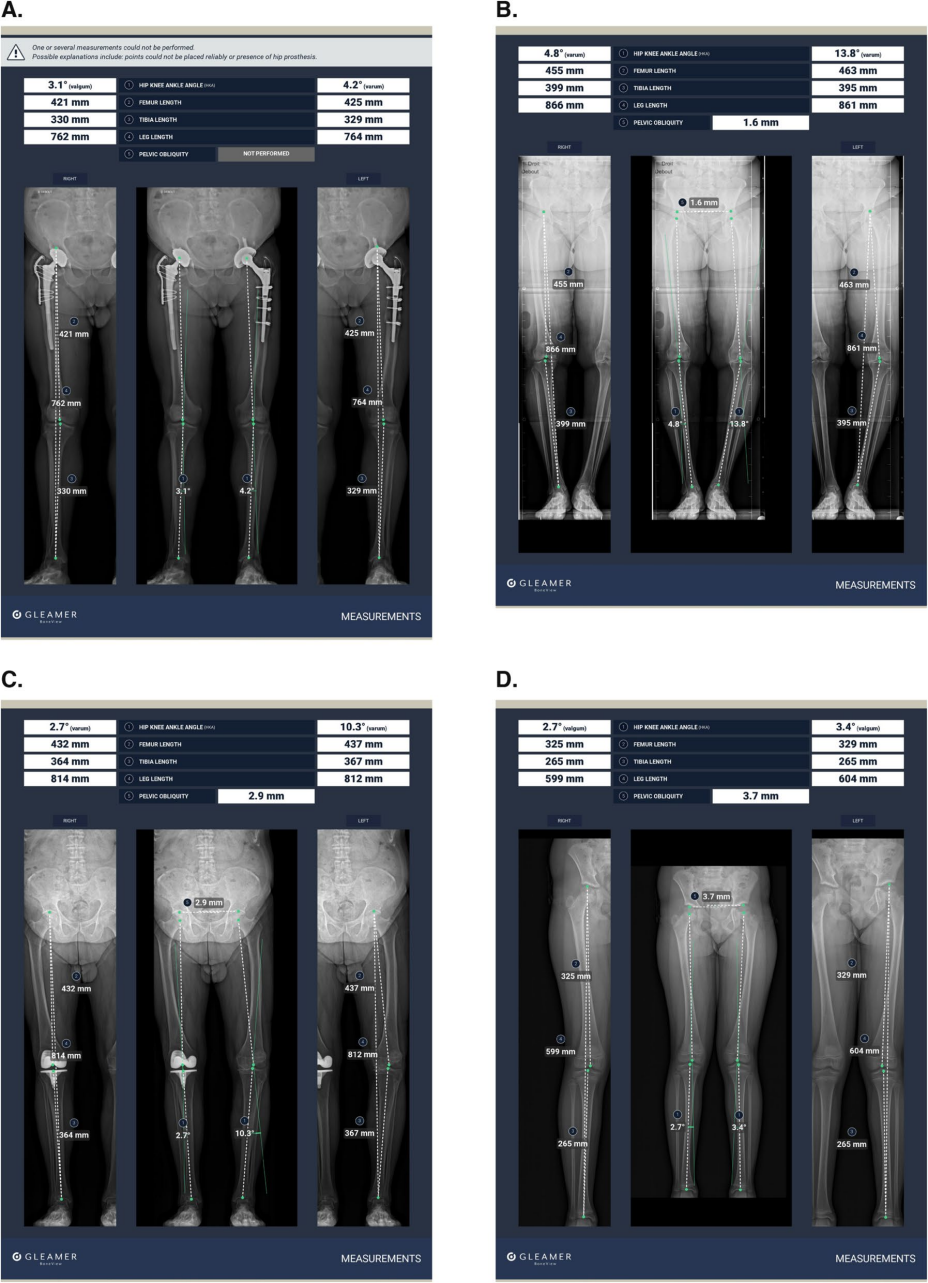
Examples of radiographs processed by the AI algorithm. **A** A 60-year-old male patient with bilateral hip prostheses. Note that pelvic obliquity is not processed by the AI algorithm if the patient has a hip prosthesis. **B** A 73-year-old female patient with bilateral genu varum deformity. **C** A 78-year-old male patient with a right knee prosthesis and severe left genu varum deformity. **D** An 8-year-old girl with genu valgum deformity

Figure 4 illustrates BoneMetrics outcomes in a patient with a hip prosthesis, a patient with genu varum, a patient with a knee prosthesis, and a pediatric patient with genu valgum.

### Inter-reader and intra-reader agreement

ICC estimates between the two expert radiologists who established the ground truth showed excellent agreement across all measurements, with ICCs ≥ 0.99. The intra-reader reliability also exhibited excellent agreement, with ICCs ≥ 0.98 (Table 5).

## Discussion

Our study investigated the performance of BoneMetrics for automated leg measurements on anteroposterior full-leg standing radiographs. It revealed excellent agreement between the AI algorithm and the gold standard for all parameters (ICC ≥ 0.97). Results demonstrated the high accuracy of the AI algorithm with a MAE equal to 0.3° for the HKA angle and less than 1.5 mm for the pelvic obliquity, top leg length, center leg length, top femoral length, center femoral length, and tibial length. Clinically meaningful differences in most previous reports have been defined as deviations greater than 2° for angle measurements and superior to 5 mm for length measurements [31, 41–43]. In this study, discrepancies between automated measurements and ground truth manual measurements were far below these thresholds. Additionally, Bland–Altman plots highlighted the absence of strong bias associated with the automated measurements.

The MAE values between AI and ground truth measurements of the HKA angle range from 0.20° to 0.58° in the literature and are comparable to the value of 0.30° found in this study [31, 32, 42, 44]. For leg length measurements, our MAE values spanned from 0.95 mm to 1.46 mm. Reported differences between AI and manual measurements vary rather widely, with values from 0.10 mm [32] up to 5 mm [31]. Interestingly, lengths measured from the top of the femoral head deviated less from the ground truth (MAE = 0.95–1.03 mm) than lengths measured from the center of the femoral head (MAE = 1.23–1.46 mm). The latter findings may be explained by the poorer reproducibility in determining the center compared with the top of the femoral head, and they suggest that full-leg and femoral length measurements with this AI algorithm are more accurate using the top of the femoral head.

The AI algorithm provided reliable measurements across a very diverse patient population. The AI demonstrated equal accuracy in both pediatric and adult patients. This finding is particularly significant as undiagnosed lower limb malalignment in children can result in abnormal motor development and gait [45]. On radiographs of patients with hip implants, measurements were comparable to those of patients without implants for five out of seven parameters. Interestingly, in patients with hip implants, the AI made larger errors in measuring the center femoral length but smaller errors in measuring the center leg length. This discrepancy could be due to the higher likelihood of these patients also having knee osteoarthritis. Research has shown that knee osteoarthritis is associated with intercondylar notch stenosis, which could complicate the accurate placement of the center of the femoral intercondylar notch, a necessary landmark in measuring femoral length [46, 47]. Regarding patients with knee implants, the AI algorithm made larger errors in measuring tibial length on radiographs with knee implants than on those without. This finding suggests that positioning a landmark at the center of the knee constitutes a less reproducible task in patients with a knee prosthesis. Interestingly, EOS imaging enhanced the accuracy of HKA angle measurements in comparison with conventional radiography and did not have a significant impact on the other parameters, suggesting that AI measurements are robust to imaging modality. These findings are particularly relevant given that EOS imaging offers multiple advantages over conventional radiography, including the absence of stitching artifacts and reduced radiation exposure [48].

Our study has several strengths. First, this study is the first, to the best of our knowledge, to examine the performance of an AI algorithm on total leg and femoral length measurements from two distinct landmark points, as most studies to date have focused predominantly on angle measurements [33, 41, 42, 44, 49–51]. Our results confirm the feasibility of automated measurements not only for angles but also for a comprehensive set of leg length measurements.

Additionally, the AI software exhibited highly reliable measurements of the HKA angle, even in patients with malalignment including genu varum and genu valgum. Although the MAE was significantly different between patients with genu varum (0.33°) and those without (0.30°), it remained well below clinically meaningful thresholds. A key strength of this study also pertains to the generalizability of findings, as data were collected consecutively across various centers, manufacturers, and imaging modalities. Future investigations should further expand patient diversity to test the performance of the AI on images with skeletal abnormalities or different implant configurations. Finally, it is important to highlight that the issue of data dependencies was alleviated by employing a mixed-effects approach to the Bland–Altman analysis.

There are limitations to our study. First, the retrospective design means that the performance of the AI algorithm in a clinical setting and its impact on physicians’ workflow and patient care could not be evaluated. Moreover, the small dataset may not have fully captured the diversity of patient demographics and radiographic conditions, thus restricting the AI’s generalizability. The study did not examine key leg alignment parameters, such as the medial proximal tibial angle, lateral distal femoral angle, and joint line convergence angle, which play an essential role in clinical decision-making and surgical planning. Future studies should evaluate the performance of the AI on these specific measurements. Another limitation is that some landmark points were positioned with low confidence levels (< 50%) by the AI algorithm for reasons that are difficult to disentangle. Finally, the dataset exhibited a bias toward radiographs of patients with malalignment, resulting in an under-representation of healthy lower limbs.

In conclusion, our study underscores the reliability of a commercially available AI-based software in automating measurements on anteroposterior full-leg standing radiographs. The AI algorithm exhibited good accuracy in computing critical lower limb parameters including the HKA angle, pelvic obliquity, total leg length (measured from two distinct landmark points), femoral leg length (measured from two distinct landmark points), and tibial length. The results closely align with findings reported in similar previous studies. Future work should focus on whether the integration of such an AI tool into clinical workflows could alleviate physicians’ workload by facilitating laborious and time-consuming tasks. It should also evaluate the clinical implications of automated measurements, particularly for preventive medicine, surgical planning, and postoperative monitoring.

## Data Availability

The datasets used and analyzed for the current study are available from the corresponding author upon reasonable request. The dataset used for developing the AI model, as well as the AI model itself, are components of proprietary software and are therefore not publicly available.

## Abbreviations

AI: Artificial intelligence
Center femoral length: Femoral length from the center of the femoral head
Center leg length: Leg length from the center of the femoral head
HKA: Hip–knee–ankle
ICC: Intraclass correlation coefficient
MAE: Mean absolute error
RMSE: Root mean square error
Top femoral length: Femoral length from the top of the femoral head
Top leg length: Leg length from the top of the femoral head
